# CircRNA NALCN acts as an miR-493-3p sponge to regulate PTEN expression and inhibit glioma progression

**DOI:** 10.1186/s12935-021-02001-y

**Published:** 2021-06-10

**Authors:** Yi Liu, Simin Chen, Gang Peng, Yiwei Liao, Xuegong Fan, Zuping Zhang, Chenfu Shen

**Affiliations:** 1 Department of Neurosurgery, Xiangya Hospital, Central South University, Changsha, Hunan China; 2 School of Basic Medicine, Central South University, Changsha, Hunan China; 3 Hunan Key Laboratory of Viral Hepatitis, Xiangya Hospital, Central South University, Changsha, Hunan China

**Keywords:** Glioma, Prognosis, circNALCN, miR-493-3p, PTEN, ceRNA

## Abstract

**Background:**

An increasing number of studies have shown that circular RNAs (circRNAs) play important roles in the regulation of tumor progression. Therefore, we explored the expression characteristics, function, and related mechanism of the newly identified circNALCN in glioma.

**Methods:**

RNA sequencing was used to analyze the expression profiles of circRNAs in brain tissue from five glioma cases and four normal controls. Quantitative real-time polymerase chain reaction was implemented to examine the levels of circNALCN, miR-493-3p, and phosphatase and tensin homolog (PTEN). Cell counting kit 8 assays were performed to analyze cell proliferation, and cell migration was assessed by the wound healing test and Transwell assay. Dual-luciferase reporter, fluorescence in situ hybridization, and RNA pulldown assays were performed to confirm the role of circNALCN as an miR-493-3p sponge, weakening the inhibitory effect of miR-493-3p on target PTEN expression.

**Results:**

The downregulated expression of circNALCN was observed in both glioma tissues and cell lines. CircNALCN expression was negatively correlated with World Health Organization grade and overall survival in patients with glioma. Functionally, the overexpression of circNALCN significantly inhibited the proliferation and migration of glioma cells, whereas miR-493-3p mimics counteracted these effects. The mechanistic analysis demonstrated that circNALCN acted as a competing endogenous RNA for miR-493-3p to relieve the repressive effects of miR-493-3p on its target, PTEN, suppressing glioma tumorigenesis.

**Conclusions:**

CircNALCN inhibits the progression of glioma through the miR-493-3p/PTEN axis, providing a developable biomarker and therapeutic target for glioma patients.

**Supplementary Information:**

The online version contains supplementary material available at 10.1186/s12935-021-02001-y.

## Background

Gliomas account for approximately 30% of all intracranial tumors and are the most common primary brain tumors [1]. Unfortunately, most gliomas (approximately 80%) are malignant in nature, with a worldwide incidence of 3.9 per 100,000 in men and 3.1 per 100,000 in women [2]. Currently, comprehensive treatment methods, including surgery, radiotherapy, chemotherapy, and immunotherapy, are the primary options applied in the clinic. However, due to the unclear pathogenesis of glioma and the lack of specific treatment targets, the overall effect is often unsatisfactory, resulting in a median survival time of only 15–16 months among patients with World Health Organization (WHO) Grade IV glioblastoma [3]. In recent years, gene therapy for glioma has been widely studied, but no specific targets or mechanisms have been clearly identified. Therefore, the treatment and prognosis of glioma are likely to benefit greatly from explorations of the underlying molecular mechanism of glioma.

Circular RNAs (circRNAs) are a newly discovered class of endogenous, non-coding RNA found in eukaryotes. CircRNAs are typically comprised of exons that form covalently closed, continuous loops and contain typical splicing sites [4, 5]. Traditionally, circRNAs were considered to be meaningless by-products of gene rearrangements and mRNA splicing [6]. However, due to advances in high-throughput sequencing and bioinformatics analyses, emerging studies have demonstrated that circRNAs are evolutionarily conserved, stable, and richly expressed, with specific expression patterns across different cells and developmental stages [7]. In addition, circRNAs have been associated with a variety of functions, including acting as a microRNA (miRNA) sponges, binding to RNA binding proteins (RBPs), and participating in protein translation [8, 9].

By analyzing non-coding RNAs expressed in 29 different stages of nerves and tissue, AgnieszkaRybak-Wolf identified the rich and specific expression of circRNAs throughout the brain, which showed a high degree of stability and incompatibility compared with related linear mRNAs [10], indicating that circRNAs may be involved in neurodifferentiation processes and the development of neurological diseases. However, the abundance of circRNAs found in glioma specimens is lower than that in normal samples, which prompted us to consider the role played by circRNAs in glioma progression and potential clinical applications [11]. An in-depth understanding of the role and mechanism of circRNAs in glioma will be helpful for developing new detection methods and effective treatments.

MicroRNAs (miRNAs) are a type of ubiquitous and conserved non-coding small RNA, which act as negative gene regulators that inhibit the expression of target genes [12]. They are involved in a wide range of biological processes associated with cancer, including proliferation, invasion, differentiation, and apoptosis [13]. Increasing evidence has suggested that circRNAs play important roles in tumorigenesis and cancer development by regulating miRNAs [14]. For example, circHIPK3 can bind to multiple miRNAs, including miR-654 and miR-124-3p, and several studies have demonstrated that circHIPK3 targets miR-654, which targets insulin-like growth factor 2 mRNA binding protein 3 (IGF2BP3), suggesting that circHIPK3 promotes glioma progression by regulating the miR-654/IGF2BP3 axis [15]. Another study showed that circ-HIPK3 promotes the proliferation and invasion of glioma cells by acting as a sponge for miR-124-3p, resulting in the upregulation of signal transducer and activator of transcription 3 (STAT3) [16]. However, there may be other circRNAs that are regulated by the ceRNA network in the occurrence and development of gliomas have not been discovered.

Several studies have shown that phosphatase and tensin homolog (PTEN) is a multifunctional tumor suppressor that contains a catalytic domain and a tensin-like domain [17, 18]. PTEN plays an important role in the regulation of cell proliferation, apoptosis, and tumor invasion. The results of clinical studies examining high-grade gliomas have shown that changes in PTEN gene expression are associated with poor prognosis and may affect the response to specific treatments [19, 20]. One mechanism involved in the regulation of PTEN expression is miRNA-mediated regulation [21, 22].

Based on RNA sequencing (RNA-seq) results, we first identified a novel glioma-related circRNA, termed circNALCN (circBase ID: hsa_circ_0099761), which is derived from exons 2 to 7 of the sodium leak channel, non-selective (*NALCN*) gene. We found that circNALCN has not yet mentioned its role in tumors (including glioma) and other diseases. To obtain insights into the function and underlying molecular mechanisms through which circNALCN participates in the development and progression of glioma, the clinical significance of circNALCN was explored in glioma tissues. CircNALCN was found to be downregulated in glioma tissues and acted as a sponge for miR-493-3p, affecting the expression of PTEN to modulate glioma tumorigenesis and progression. Collectively, our findings suggest that circNALCN can be used as a prognostic predictor and therapeutic target in glioma.

## Methods

### Sequencing data analysis

High-throughput sequencing was performed on five cases of glioblastoma and four cases of normal brain tissue, and the samples were processed and sequenced by Lc-Bio Technologie (HangZhou, China). The results were processed with the edgeR software package (https://www.Bioconductor.org/). Significant differences in circRNAs and miRNAs expression were screened to identify those with log_2_ (fold-change) ≥ 2 and P < 0.05. The results showed significant differences between circRNA and miRNA expression levels between glioblastoma and normal cases.

### Patient tissue samples

In our study, 76 cases of glioma and 17 normal tissues were collected from August 2018 to May 2020 at Xiangya Hospital. All patients underwent surgical resection of glioma or non-tumor operations, such as craniocerebral trauma, which were performed in accordance with the Helsinki Declaration. After surgical resection, all specimens were frozen in liquid nitrogen and stored at − 80 °C until RNA was extracted. The study was approved by the Ethics Committee of Xiangya Hospital of Central South University, and informed consent was obtained from all patients.

### Cell culture

Human glioma SF126 cells were obtained from the Cell Research Institute of Peking Union Medical College (Peking, China). Human U251 and U87 glioma cells were obtained from the American Type Culture Collection (ATCC). The normal glial cell line HEB was purchased from the Cell Bank of the Type Culture Collection of the Chinese Academy of Sciences (Shanghai, China). The cells were cultured in Dulbecco’s modified Eagle medium (DMEM) containing 10% fetal bovine serum (FBS) and 100 U/mL penicillin/streptomycin (all from Gibco, Carlsbad, CA, USA) at 37 °C in a humidified incubator containing 5% CO_2_.

### RNA extraction and quantitative real-time polymerase reaction (qRT-PCR)

Total RNA was extracted from glioma and normal tissues and cell lines using TRIzol Reagent (Takara, Japan), and genomic DNA (gDNA) was isolated with FastPure DNA Isolation (Vazyme, China), according to the manufacturer’s instructions. The concentration and purity of RNA samples were measured using a Nanodrop 2000 (Thermo Fisher Scientific, USA). For circRNA and mRNA, reverse transcriptions were performed using the PrimeScript RT Master Mix (Takara, Japan) with random primers. For miRNA, reverse transcriptions were performed using the PrimeScript RT Reagent Kit (Takara, Japan) with specific stem-loop primers. cDNA amplification was performed using Hieff^®^ qPCR SYBR Green Master Mix (Low Rox Plus, YeSen, Shanghai, China) using an ABI Prism 7500 sequence detection system (Applied Biosystems, USA). PCR amplification conditions were as follows: initial denaturation for 30 s at 95 °C, and 40 cycles of 10 s at 95 °C, followed by 30 s at 60 °C. Glyceraldehyde 3-phosphate dehydrogenase (GAPDH) and U6 were used as internal controls, and each sample was measured three times. The relative quantification of circRNA, miRNA, and mRNA expression levels were compared to the expression level of the internal control and analyzed using the 2^−ΔΔCT^ method. Divergent primers were used to detect the back splice junction of circRNAs, and convergent primers were used to detect linear mRNA. All primers used in this study are listed in Table 1.

**Table 1:** Primer sequences used for qRT-PCR

Name	Primer	Sequence (5ʹ-3ʹ)
circNALCN	Forward primer Reverse primer	AGATCTGGGACTTAGCAGGC TGAGCAAGCACAAAACCACA
Linear NALCN	Forward primer Reverse primer	CTGAGCCGCCGTAGACTG CATGAGGAACACCCAGCCTT
miR-493-3p	RT primer	GTCGTATCCAGTGCAGGGTCCGAGGTATTCGCACTGGATACGACCCTGGC
	Forward primer Reverse primer	GCGCGTGAAGGTCTACTGTGT AGTGCAGGGTCCGAGGTATT
PTEN	Forward primer Reverse primer	TGTGGTCTGCCAGCTAAAGG CGGCTGAGGGAACTCAAAGT
GAPDH	Forward primer Reverse primer	GTCTCCTCTGACTTCAACAGCG ACCACCCTGTTGCTGTAGCCAA
U6	Forward primer Reverse primer	AACGCTTCACGAATTTGCGT CTCGCTTCGGCAGCACA

### Nucleic acid electrophoresis

cDNA and gDNA amplification products were detected by 2% agarose gel electrophoresis in tris-acetate-EDTA (TAE) buffer. DNA was separated by 120 V electrophoresis for 25 min. DNA sizes were compared against Marker L (50–5000 bp) (Sango Biotech, China). The bands were detected by ultraviolet light.

### RNase R treatment

The RNAs (1 µg) from U87 and U251 cells were treated with RNase R (2 U/µg, GeneSeed, Guangzhou, China) and incubated at 37 °C for 30 min, followed by inactivation by 10 min at 70 °C. The treated RNAs were reverse transcribed with divergent primers or convergent primers and detected by qRT-PCR assay.

### Cell transfection

CircNALCN small interfering RNA (si-circNALCN), miR-493-3p mimics, miR-493-3p inhibitor, negative control (NC) mimics, and NC inhibitor were all designed and synthesized by RiboBio (Guangzhou, China). The circNALCN overexpression plasmid (pcDNA3.1-circNALCN), PTEN overexpression plasmid (pcDNA3.1-PTEN), and pcDNA3.1-NC were synthesized by Genscript (Nanjing, China). U87 and U251 cells were inoculated in 6-well plates and cultured to 60–70% confluence, and si-circNALCN, miR-493-3p mimic, miR-493-3p inhibitor and control RNA (Ribio, Guangzhou, China) were transfected into U87 and U251 cells with a final concentration of 20 uM Lipofectamine^®^ 2000 (Invitrogen; Thermo Fisher Scientific, Inc.) for continuous 6 h. Similarly, 2000 ng pcDNA3.1-circNALCN, pcDNA3.1-PTEN and pcDNA3.1-NC were also transfected into U87 and U251 cells. The transfected cells were incubated at 37 °C and 5% CO_2_ for 24 h, and the transfection efficiency was detected by reverse transcription-polymerase chain reaction (RT-PCR).

### Luciferase reporter assay

The sequences of circNALCN and PTEN-3ʹUTR and their corresponding mutation were designed, synthesized and inserted into luciferase reporter vector pmirGLO (Promega, USA), termed circNALCN-WT, circNALCN-MUT, PTEN-WT and PTEN-MUT, respectively. All luciferase reporter vectors were synthesized by GenScript (Nanjing, China). U87 and U251 cells were inoculated in a 96-well plate to achieve 70% confluence. The 20 uM miR-493-3p mimics and NC mimics were cotransfected with 100 ng circNALCN-WT, circNALCN-MUT, PTEN-WT, and PTEN-MUT into U87 and U251 with lipofectamine 2000. Then, the relative luciferase activity was examined using a Dual-Luciferase Assay Kit (Promega, USA), according to the manufacturer’s protocol.

### Fluorescence in situ hybridization (FISH)

Fluorescence in situ hybridization (FISH) assay was performed to observe the locations of circNALCN and miR-493-3p expression in U87 and U251 cells. Fluoresceine amidite (FAM)-labeled circNALCN probes and cyanine-3 (Cy3)-labeled miR-493-3p were designed and synthesized by Servicebio (Wuhan, China). Hybridization was performed overnight using the circNALCN and miR-493-3p probes, according to the manufacturer’s instructions. The images were acquired on a Zeiss LSM710 Laser-Scanning Confocal Microscope (Zeiss Instrument Inc., Germany). The sequences of circNALCN and miR-493-3p that were probed during FISH are listed in Additional file 1: Table S1.

### RNA pulldown assays

U251 and U87 cells (4 × 10^7^) were collected, fixed with 1% formaldehyde, and add cell lysate (BersinBio, Guangzhou, China) on ice. RiboBio (Guangzhou, China) designed and synthesized the circNALCN probe, PTEN probe, and NC biotin probe. RNA Antisense Purification (RAP) kit was purchased from bersinbio (Guangzhou, China). According to the manufacturer’s instructions, the circNALCN, PTEN and NC biotin probes were added to the cell lysate, hybridized at 37 °C for 30 min, denatured at 50 °C for 5 min, hybridized again at 37 °C for 120 min, and incubated with streptomyces and magnetic beads (BersinBio, Guangzhou, China) at 25 °C for 30 min. RNA complexes bound to magnetic biotin beads were eluted using RNA elution buffer, and qRT-PCR was performed to analyze the relative expression levels of miR-493-3p.

### Cell counting kit-8 proliferation assay

U87 and U251 cells were plated at 8 × 10^3^ cells per well of a 96-well plate. A 10 µl volume of the CCK-8 reagent (Yisheng, Shanghai, China) was directly added to the culture medium at specified times (0, 24, 48, 72 and 96 h). The cells were incubated with the CCK-8 reagent for 1 h at 37 °C, and the optional density (OD) was measured at 450 nm using a miniature tablet reader (BioTek Instruments, USA).

### Wound healing assays

U87 and U251 cells were cultured in a 6-well plate until a cell monolayer was formed. The monolayer was subsequently scratched using a 10 µl pipette tip. Cell migration images were taken at 0 and 36 h after injury in four high-power fields. After 36 h of measurement, the wound distance was reduced to the standard, normalized against the 0 h control, and expressed as relative mobility.

### Transwell migration assays

Transwell analysis was performed as described by the manufacturer. U87 and U251 cells were inoculated in the upper compartment in 100 µl of serum-free medium. The bottom chamber of the Transwell chamber (Corning, USA) was filled with 600 µl medium and 10% FBS as a glioma cell chemoattractant. After incubation for 24 h, the upper chamber was fixed and stained with crystal violet (Kaigen, China) for 15 min. Images were taken from five different fields of vision, and the cells were counted.

### Western blot

Cells were lysed in radioimmunoprecipitation assay lysis buffer (RIPA, Beyotime, China). The protein was prepared and quantified using bicinchoninic acid (BCA) analysis (Beyotime, China). Equal amounts of protein were separated by 10% sodium dodecyl sulfide-polyacrylamide gel electrophoresis (SDS-PAGE) and transferred onto polyvinylidene fluoride (PVDF) membranes (Millipore, MA, USA). Membranes were blocked with 5% skim milk powder and incubated with the primary antibodies anti-PTEN (1:1000, Bioworld Technology Inc.) and anti-GAPDH (1:10,000, Bioworld Technology Inc.) at 4 °C overnight. The membranes were incubated with a secondary antibody (1:10,000, Bioworld Technology Inc.) for 1 h in room temperature. Finally, the proteins were visualized using enhanced chemiluminescence (ECL) reagent (Yisheng, Shanghai, China), and the relevant data were analyzed by ImageLab software.

### Statistical analysis

Statistical analysis was performed using GraphPad Prism 8, and Student’s t-test and one-way analysis of variance (ANOVA) were used to determine significant differences between two or more groups. Pearson’s correlation coefficient was used to perform correlation analyses. The results are expressed as the mean ± standard deviation (mean ± SD). The overall survival (OS) curve was calculated by Kaplan–Meier analysis, and the log-rank test was performed. Differences were considered significant at P < 0.05.

## Results

### circNALCN (hsa_circ_0099761) is expressed at low levels in gliomas

**Fig. 1:**
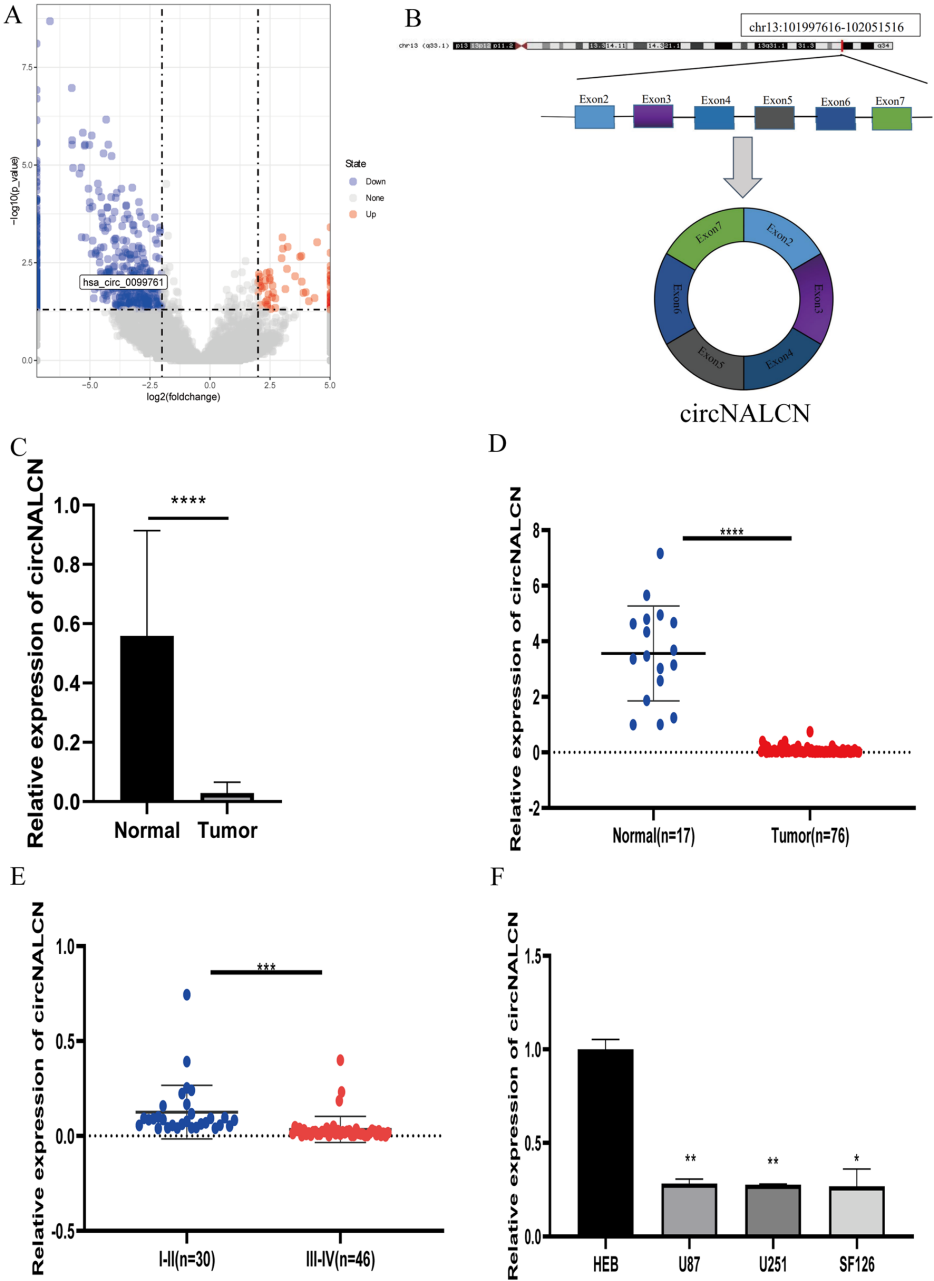
Identification and validation of differential circNALCN expression in glioma tissues and cells. **A** Volcano plot of differentially expressed circRNAs. The blue dots represent downregulated circRNAs, whereas the red dots represent upregulated circRNAs in glioma compared with normal tissue. circNALCN is labeled as hsa_circ_0099761. **B** Schematic showing the circularization of NALCN exons 2–7 in the formation of circNALCN. **C** qRT-PCR verified that the expression level of circNALCN in glioma tissues was lower than that in normal brain tissues in the sequenced specimens. **D** circNALCN expression was detected via qRT-PCR in 76 cases of glioma tissues and 17 cases of normal brain tissues. **E** circNALCN is expressed in WHO Grade I–II and WHO Grade III–IV glioma. **F** circNALCN expression was detected via qRT-PCR in various glioma cell lines (U87, U251, and SF126) and human normal glial cells (HEB). Student’s t-test, *P < 0.05, **P < 0.01, ***P < 0.001, ****P < 0.0001

We analyzed five cases of glioblastoma tissues and four cases of normal brain tissues by high-throughput RNA-seq. A total of 20,204 circRNAs were sequenced using edgeR packets to perform difference analyses of glioma tissue and normal brain tissue. Based on the condition of |log_2_ (fold change(FC)) >  = 2| and P < 0.05, 652 differentially expressed circRNAs (DEcircRNAs), including 67 upregulated DEcircRNAs and 585 downregulated DEcircRNAs were identified. In cancers with high cell proliferation rates, circRNA proliferation has been reported to be diluted and downregulated [23]. Therefore, we focused on the significantly downregulated circRNAs. Using the resulting volcano plot, we identified a significantly downregulated circRNA (hsa_circ_0099761), named circNALCN, which is transcribed from *NALCN* on human chromosome 13 through the head–tail splicing of exons 2–7 (Fig. 1A, B). And then we found that circNALCN is a novel circRNA, that has not been reported in tumors and other diseases, and its function is not clear, so we studied the function of circNALCN in glioma cells. We verified that the available sequencing data for circNALCN and our RNA-seq data were essentially the same. Compared with normal brain tissue, circNALCN decreased in glioma tissue (Fig. 1C). To further verify whether circNALCN expression was downregulated in glioma tissues and cells, we used qRT-PCR analysis to examine circNALCN expression in 76 cases of glioma tissue and 17 cases of normal brain tissue. We found that the expression of circNALCN was significantly decreased in glioma tissues compared with the expression level in normal tissues (Fig. 1D and Table 2, P < 0.001). We also found that the expression levels of circNALCN in WHO Grade I–II gliomas were higher than that in WHO Grade III–IV gliomas (Fig. 1E, P < 0.001), indicating that circNALCN expression may be related to glioma tumor grade.

**Table 2:** Relationship between circNALCN and miR-493-3p expression and clinicopathological characteristics in 76 cases of glioma tissue

Characteristics	Case	circNALCN expression		P-value	miR-493-3p expression		P-value
		Low	High		Low	High	
All cases	76	38	38		38	38	
Age (years)				0.2499			0.0387
> 50	34	21	13		15	19	
≤ 50	42	17	25		23	19	
Sex				0.2812			0.3443
Male	38	22	16		20	18	
Female	38	16	22		18	20	
Grade				0.0004			0.0331
I–II	30	0	30		17	13	
III–IV	46	36	8		21	25	

Next, we compared circNALCN expression levels between HEB, U87, U251 and SF126 cells lines. Compared with the normal glial cell line (HEB), the expression of circNALCN is lower in glioma cells. No significant differences were observed for the expression levels of circNALCN among the various glioma cell lines (Fig. 1F). Therefore, we selected the U87 and U251 cell lines to study the downstream regulatory pathway associated with circNALCN.

### Characteristics and clinical features of circNALCN

**Fig. 2:**
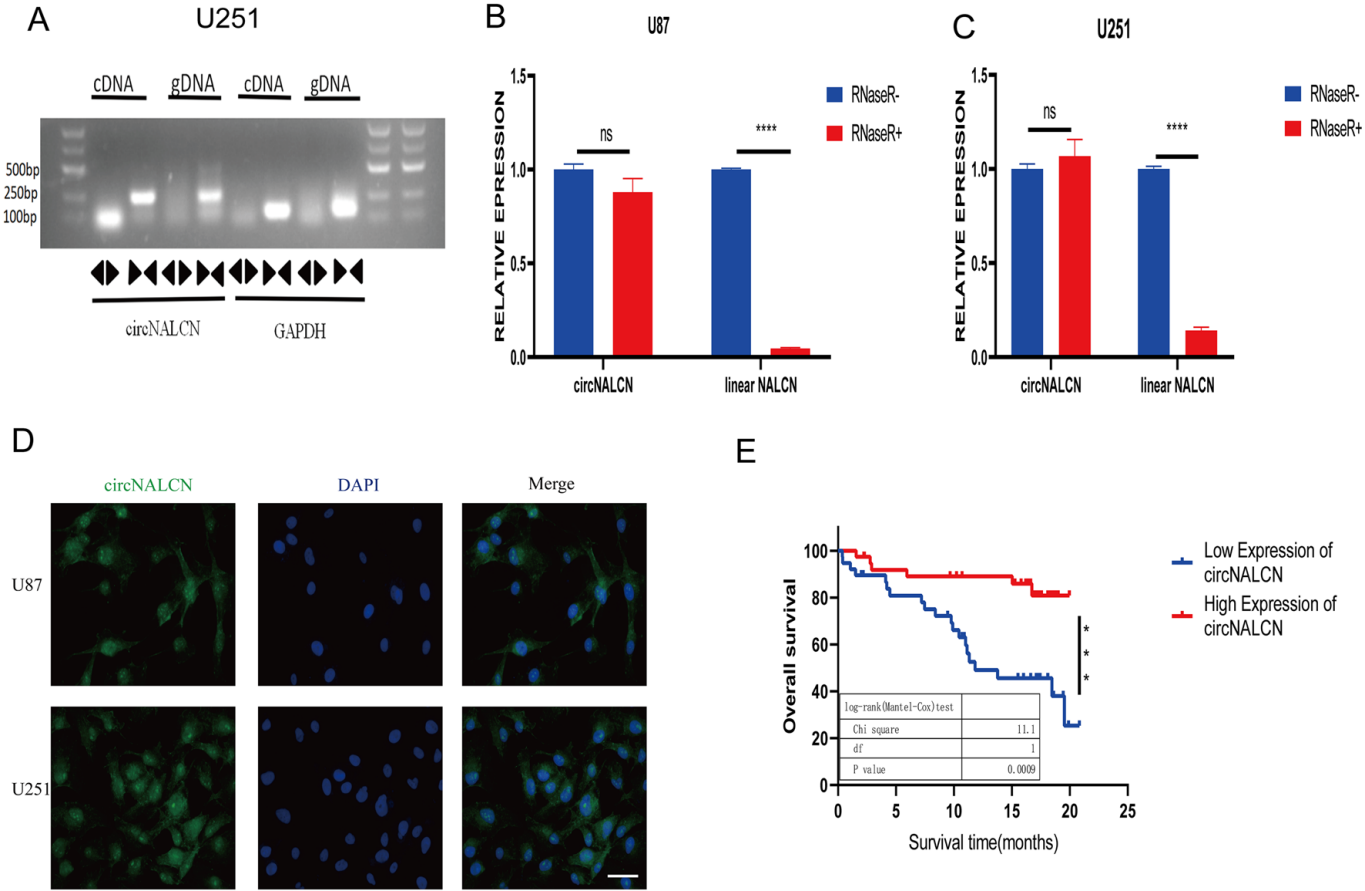
Characterization and clinical features of circNALCN. **A** Nucleic acid electrophoresis confirmed that circNALCN is a circular RNA. circNALCN was amplified by divergent primers in cDNA but not gDNA. **B**, **C** The expression of circNALCN and linear NALCN mRNA in both U87 and U251 cell lines was detected by qRT-PCR in the presence or absence of RNase R. **D** FISH assay showed that circNALCN was predominantly localized in the cytoplasm. Nuclei were stained with DAPI in blue, and cytoplasmic circNALCN were stained in green. (magnification, 400×, scale bar, 20 μm). **E** Kaplan–Meier survival curves of glioma patients with low and high circNALCN expression levels, using the median circNALCN value as a cutoff. Data are showed as the mean ± SD; ns indicates no significance, *P < 0.05, **P < 0.01, ***P < 0.001, ****P < 0.0001

CircNALCN originates from *NALCN*, located on chromosome 13. CircNALCN is formed by the splicing of exons 2–7 (101997616–102051516) from head to tail (Fig. 1B). However, head-to-tail splicing may be the result not only of trans-splicing but also of genome rearrangement. To eliminate these two possibilities, we designed special divergent and convergent primers to differentiate between circNALCN generated from cDNA and gDNA. DNA was extracted from U251 cells and analyzed by PCR and agarose gel electrophoresis. The results showed that circNALCN was only detected as cDNA, whereas no product was detected in the extracted gDNA (Fig. 2A). Stability is considered to be an important feature of circRNA [24]. The stability of circNALCN was verified by ribonuclease R (RNase R) treatment. cDNA extracted from RNase R-treated U251 and U87 cells was analyzed by PCR using specially designed divergent and convergent primers, which revealed that circNALCN could resist restriction endonuclease digestion by RNase R, whereas linear NALCN was digested by RNase R. These results suggested that circNALCN is not the result of genome rearrangement and represents a stable product. The results of the FISH analysis revealed that although circNALCN was expressed in the nucleus and cytoplasm to a certain extent, it was mainly located in the cytoplasm (Fig. 2D).

When we examined the clinical data of the examined patients, we found that the expression level of circNALCN was significantly correlated with the WHO glioma grade but not with age or sex (Fig. 1E, Table 2). In addition, the OS curve, generated using the Kaplan–Meier method, indicated that patients with low expression levels of circNALCN in glioma tissues had significantly reduced survival compared with that of patients with high levels of circNALCN in glioma tissues (P = 0.0009, Fig. 2E). These results suggested that circNALCN is a stable circRNA that may serve as a meaningful prognostic marker in glioma and warrants further study.

### CircNALCN inhibits the proliferation and migration of glioma cells

**Fig. 3:**
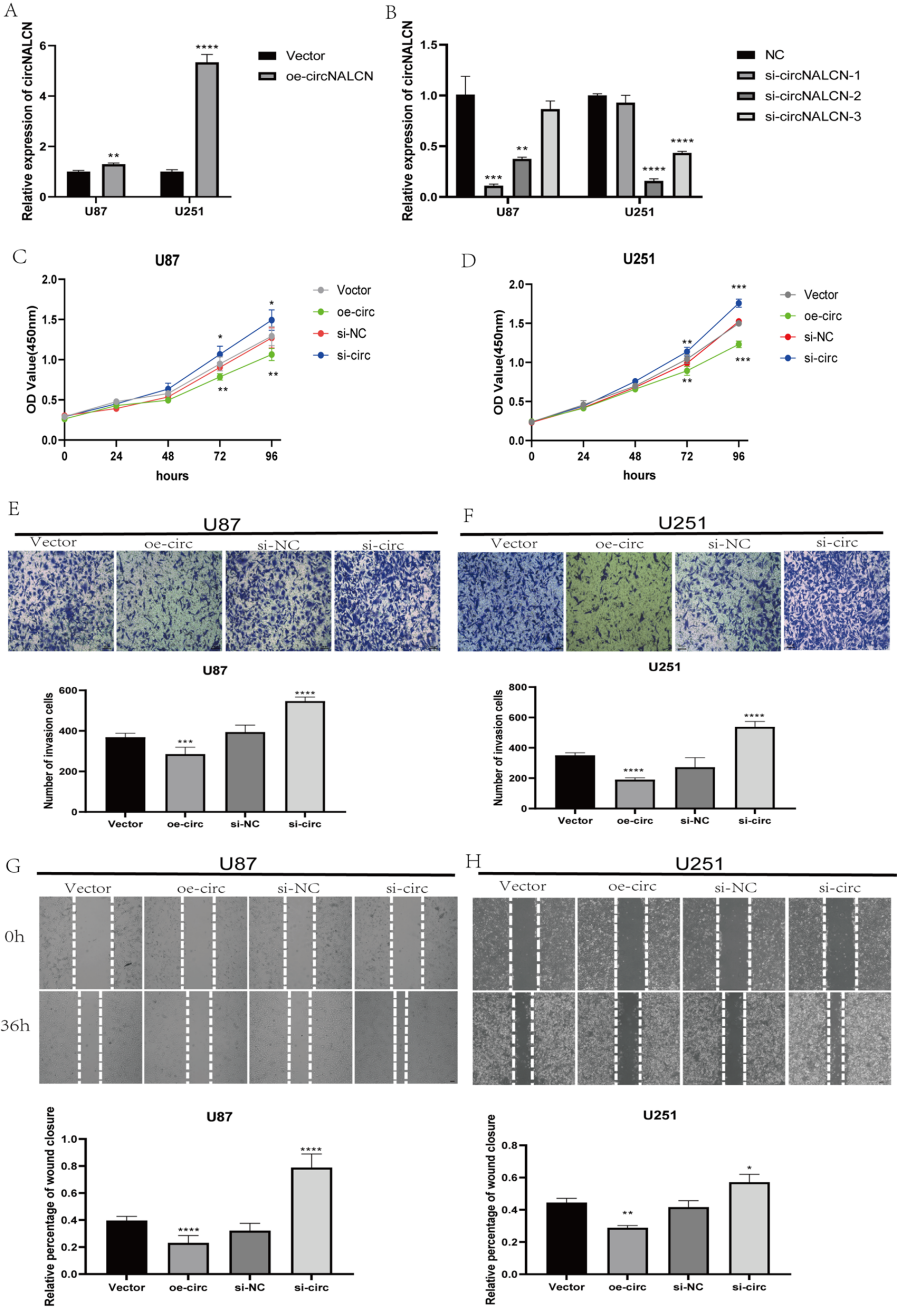
circNALCN suppresses the proliferation and migration of glioma cells. **A** qRT-PCR analysis of circNALCN expression in U87 and U251 cells overexpressing circNALCN. **B** qRT-PCR analysis of circNALCN expression in U87 and U251 treated with siRNAs. **C**, **D** CCK-8 experiment detecting the proliferation ability of U87 and U251 cells transfected with vector, oe-circNALCN, si-NC, or si-circNALCN. **E**, **F** The migration abilities of U87 and U251 cells transfected with vector, oe-circNALCN, si-NC, or si-circNALCN were detected by Transwell assay. **G**, **H** The migration abilities of U87 and U251 cells transfected with vector, oe-circNALCN, si-NC, or si-circNALCN was detected by wound healing assay. Scale bar: 100 µm. Data are presented as the mean ± SD; ns indicates no significance, *P < 0.05, **P < 0.01, ***P < 0.001, ****P < 0.0001. Vector: empty vector control; oe-circNALCN: circNALCN overexpression vector; si-NC: negative control siRNA; si-circNALCN: circNALCN siRNA

To explore the biological functions of circNALCN in glioma cells, three siRNAs were generated to target circNALCN, and a circNALCN overexpression vector was constructed. The circular transcript expression vector circNALCN was successfully constructed in U87 and U251 cells (Fig. 3A). Among the three siRNAs, si-circNALCN-2 was found to have the highest inhibition efficiency; therefore, it was selected for use in the following experiments (Fig. 3B).

CCK-8 assay revealed that circNALCN downregulation significantly enhanced cell proliferation in U87 and U251 cells, whereas circNALCN upregulation had the opposite effect. These results showed that circNALCN overexpression could inhibit the proliferation of glioma cells (Fig. 3C, D).

The effects of circNALCN on glioma cell migration was determined by the performance of the wound healing assay and the Transwell experiment. The results showed that the downregulation of circNALCN significantly enhanced the migration ability of glioma cells, whereas circNALCN upregulation significantly inhibited the migration ability of glioma cells (Fig. 3E–H). These experiments showed that circNALCN inhibited glioma cells migration.

### High expression levels of miR-493-3p in gliomas are related to poor prognosis

**Fig. 4:**
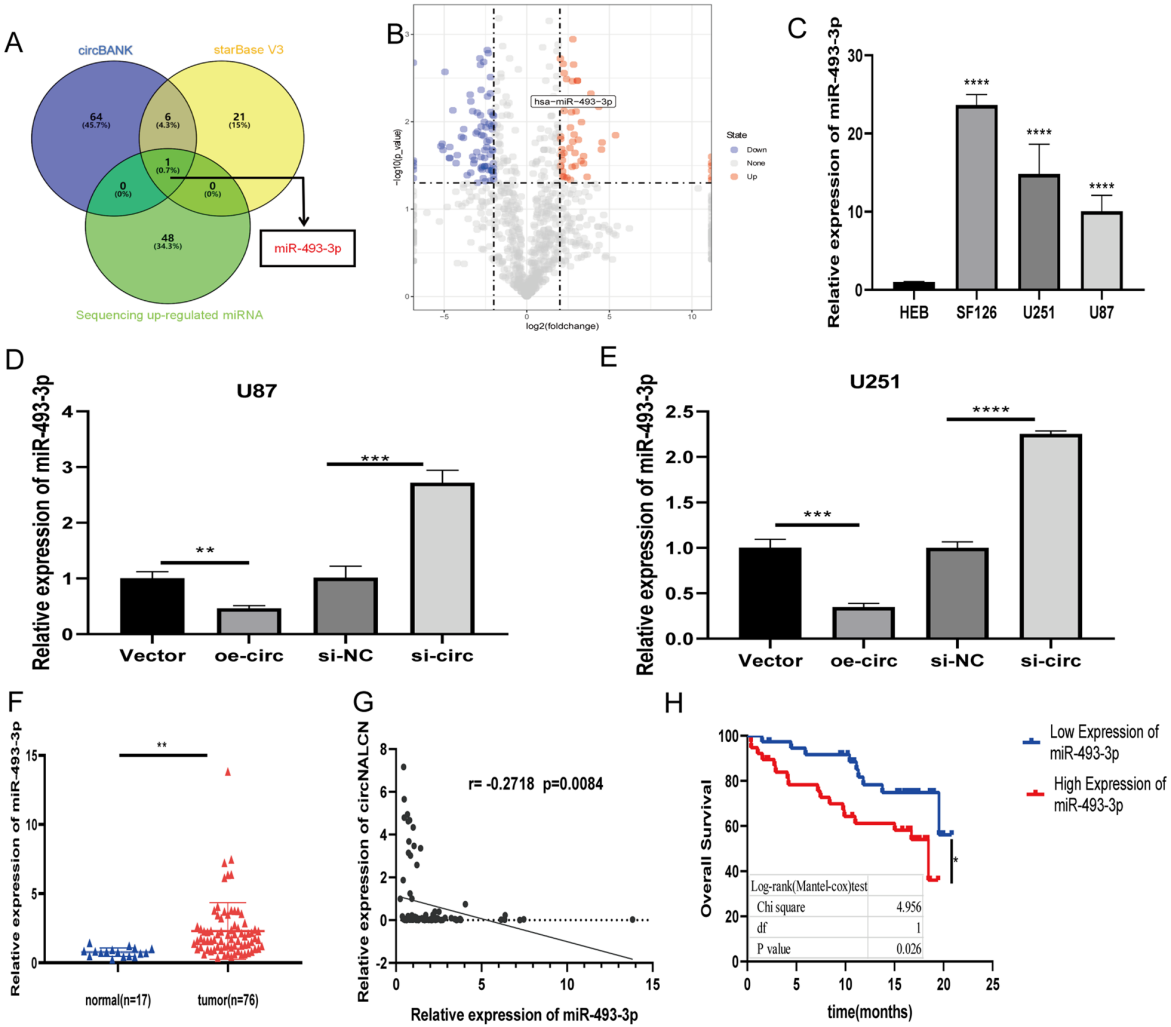
MiR-493-3p is highly expressed in glioma tissues and correlated with progression and poor prognosis. **A** Schematic illustration exhibiting the overlap among the target miRNAs for circNALCN predicted by circBank, starBase, and sequencing up-regulated miRNA data. **B** Volcano plot of differentially expressed miRNAs. The blue dots represent downregulated miRNAs, whereas the red dots represent upregulated miRNAs in glioma compared with normal tissue. MiRNA is labeled as miR-493-3p. **C** miR-493-3p expression was detected via qRT-PCR in various glioma cell lines (U87, U251 and SF126) and human normal glial cell (HEB). **D**, **E** Relative expression levels of miR-493-3p in U87 cells (**D**) and U251 cells (**E**). **F** qRT-PCR was used to detect the relative expression of miR-493-3p in glioma tissues (n = 76) and normal brain tissues (n = 17). **G** Pearson’s correlation analysis between circNALCN and miR-493-3p expression based on glioma tissues. **H** Kaplan Meier survival analysis showed that the OS of glioma patients with high miR-493-3p expression was lower than those with low miR-493-3p expression. *P < 0.05, **P < 0.01, ***P < 0.001, ****P < 0.0001

According to the theory of competitive endogenous RNAs (ceRNAs), circRNAs act as miRNA sponges to regulate miRNA expression [25]. Because circNALCN is primarily located in the cytoplasm and shows significant stability, we further explored whether circNALCN suppresses the biological behavior of gliomas by absorbing miRNAs. We used miRNA target prediction tools, including circBank, starBase, and sequencing up-regulated miRNA data, to predict potential targets for circNALCN (Fig. 4A). We sequenced a total of 1138 miRNAs and used edgR packet forward to perform differential expression analyses of glioma tissue and normal brain tissue. Based on the conditions of |log_2_(FC) >  = 2| and P < 0.05, 130 differentially expressed miRNAs (DEmiRNAs) were identified, including 49 upregulated DEmiRNAs, and 81 down-regulated DEmiRNAs. The results of three prediction methods were combined with differences in miRNA levels obtained from the sequencing results (Fig. 4B), which identified miR-493-3P as significantly upregulated in glioma tissues. The expression level of miR-493-3P was also found to be upregulated in HEB cells compared with the three glioma cell lines. Therefore, we selected miR-493-3p for further verification (Fig. 4C). We transfected si-circNALCN and circNALCN overexpression vectors into U87 and U251 cells and observed the effects on miR-493-3p expression. The results showed that miR-493-3p expression was significantly increased in the si-circNALCN group and decreased in the circNALCN overexpression group compared with untreated cells (Fig. 4D, E). The expression levels of miR-493-3p in glioma and normal tissues were detected by qRT-PCR, which showed that miR-493-3p expression was significantly increased in glioma tissues compared with that in normal tissues (Fig. 4F). We found a negative correlation between the expression levels of circNALCN and miR-493-3p in both glioma tissues and normal tissues (Fig. 4G). These results suggested that circNALCN might serve as an miR-493-3p sponge. In addition, we analyzed the relationship between miR-493-3p expression levels and the clinical characteristics of patients with gliomas, as shown in Table 2. The expression of miR-493-3p was correlated with WHO tumor grade and age. The Kaplan–Meier survival curve showed that the expression of miR-493-3p was negatively correlated with the OS among glioma patients (Fig. 4H), suggesting that miR-493-3p may serve as a prognostic indicator.

### CircNALCN acts as an miR-493-4p sponge in glioma

**Fig. 5:**
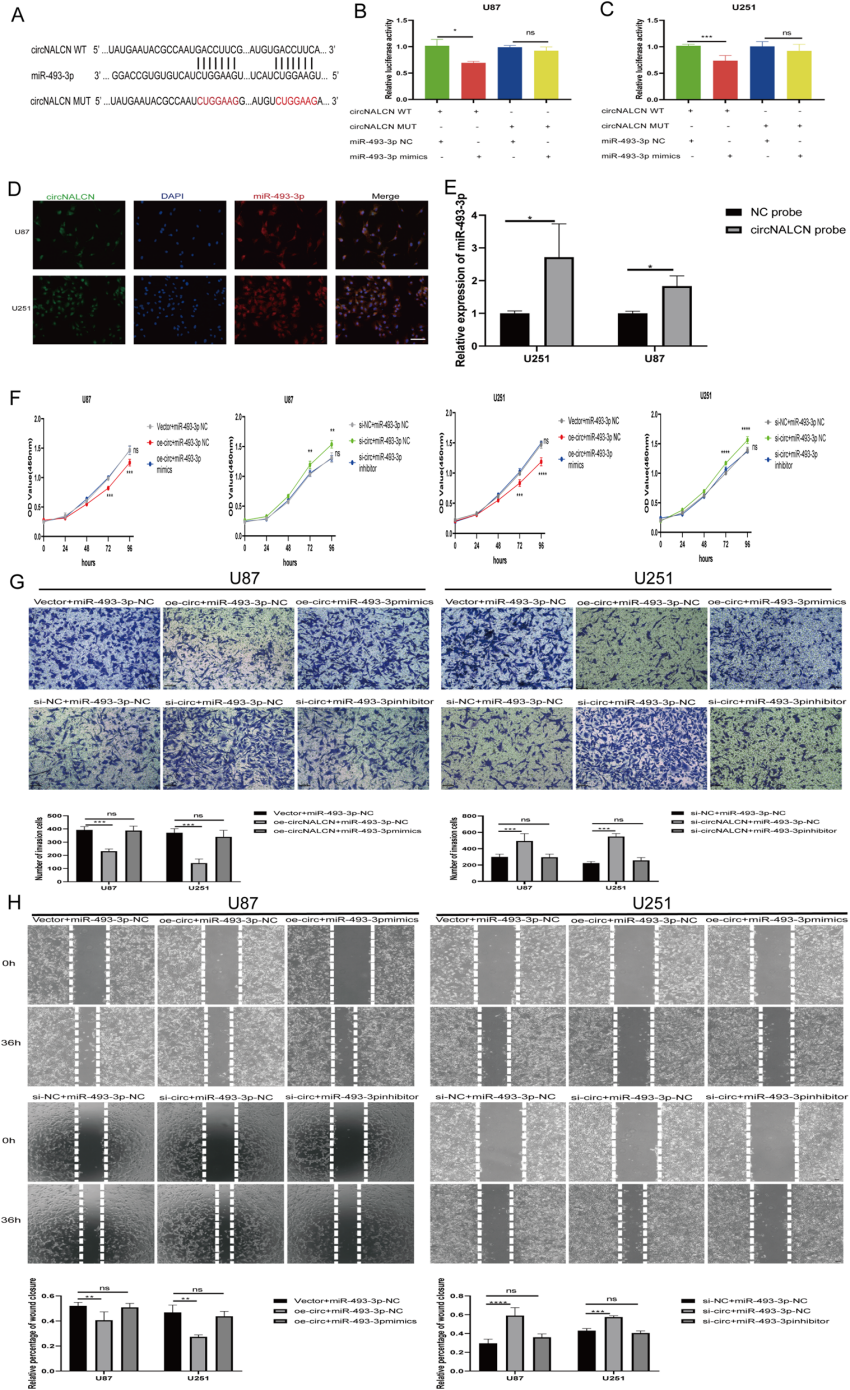
circNALCN acts as an miRNA sponge for miR-493-3p, and the antitumor effect of circNALCN in glioma can be reversed by miR-493-3p. **A** Schematic illustration of circNALCN-WT and circNALCN-MUT luciferase reporter vectors. **B**, **C** After co-transfection with circNALCN-WT or circNALCN-MUT and miR-93-3p or NC mimics, the relative luciferase activity of U87 and U251 cells were detected. **D** FISH was performed to observe the cellular location of circNALCN (green) and miR-493-3p (red) in cells (magnification, 200×, scale bar, 50 µm). **E** RNA pulldown assay was executed in U87 and U251 cells, followed by qRT-PCR to detect the expression of miR-493-3p. **F** The proliferative abilities of U87 and U251 cells transfected with vector and miR-493-3p NC, oe-circNALCN and miR-493-3p mimics, oe-circNALCN and miR-493-3p NC, si-NC and miR-493-3p NC, si-circNALCN and miR-493-3p inhibitor, and si-circNALCN and miR-493-3p NC was detected by CCK-8. **G**, **H** The migration abilities of U87 and U251 cells transfected with vector and miR-493-3p NC, oe-circNALCN and miR-493-3p mimics, oe-circNALCN and miR-493-3p NC, si-NC and miR-493-3p NC, si-circNALCN and miR-493-3p inhibitor, and si-circNALCN and miR-493-3p NC was tested by wound healing test and Transwell assay. Scale bar: 100 µm. Data are presented as the mean ± SD; ns indicates no significance. *P < 0.05, **P < 0.01, ***P < 0.001, ****P < 0.0001. Vector: empty vector control; NC: negative control oe-circNALCN: circNALCN overexpression vector; si-NC: negative control siRNA; si-circNALCN: circNALCN siRNA

To study whether circNALCN serves as an miR-493-3p sponge, double-luciferase reporter gene analysis was applied to glioma cells. The full-length circNALCN-WT sequence and a mutant sequence without the miR-493-3p binding site (circNALCN-MUT) were subcloned into the pmirGLO luciferase reporter vector (Fig. 5A). The results indicated that the luciferase activity of the circNALCN-WT group significantly decreased in the miR-493-3p mimic group compared with the NC mimic group in U87 and U251 cells (Fig. 5B, C). No significant difference in luciferase activity was observed between the miR-493-3p mimics and NC mimic group in cells transfected with the circNALCN-MUT vector. In addition, FISH assays were performed in U87 and U251 cells to observe the subcellular localization of circNALCN and miR-493-3p. In our study, circNALCN (green) and miR-493-3p (red) were both primarily located in the cytoplasm, and circNALCN and miR-493-3p colocalized, suggesting that they may interact (Fig. 5D). To further confirm the direct interaction between circNALCN and miR-493-3p, we applied an RNA pulldown assay using a biotin-labeled circNALCN probe. In both U87 and U251 cells, miR-493-3p was significantly enhanced upon circNALCN capture (approximately fourfold increase in U87 cells and a twofold increase in U251 cells; Fig. 5E).

### MiR-493-3p reverses the antitumor effects of circNALCN on glioma

Next, we designed a rescue experiment to explore whether circNALCN inhibits the malignant behavior of gliomas by interacting with miR-493-3p. U87 and U251 cells were cotransfected with si-circNALCN or circNALCN overexpression vector and miR-493-3p inhibitor or mimics. CCK-8 experiments showed that miR-493-3p mimics reversed the decrease in proliferation rate caused by the overexpression of circNALCN, whereas the miR-493-3p inhibitor attenuated the increase in the proliferation rate of glioma cells induced by circNALCN depletion (Fig. 5F). In addition, the Transwell migration assay and wound healing analysis showed that the miR-493-3p mimics group reversed the inhibition of glioma cell migration caused by circNALCN overexpression, whereas the miR-493-3p inhibitor group reversed the promotion of glioma cell migration caused by circNALCN depletion (Fig. 5G, H). These findings suggested that circNALCN inhibited the progression of gliomas by sponging miR-493-3p.

### The miR-493-3p targeting of PTEN promotes the proliferation and migration of glioma cells in vitro

**Fig. 6:**
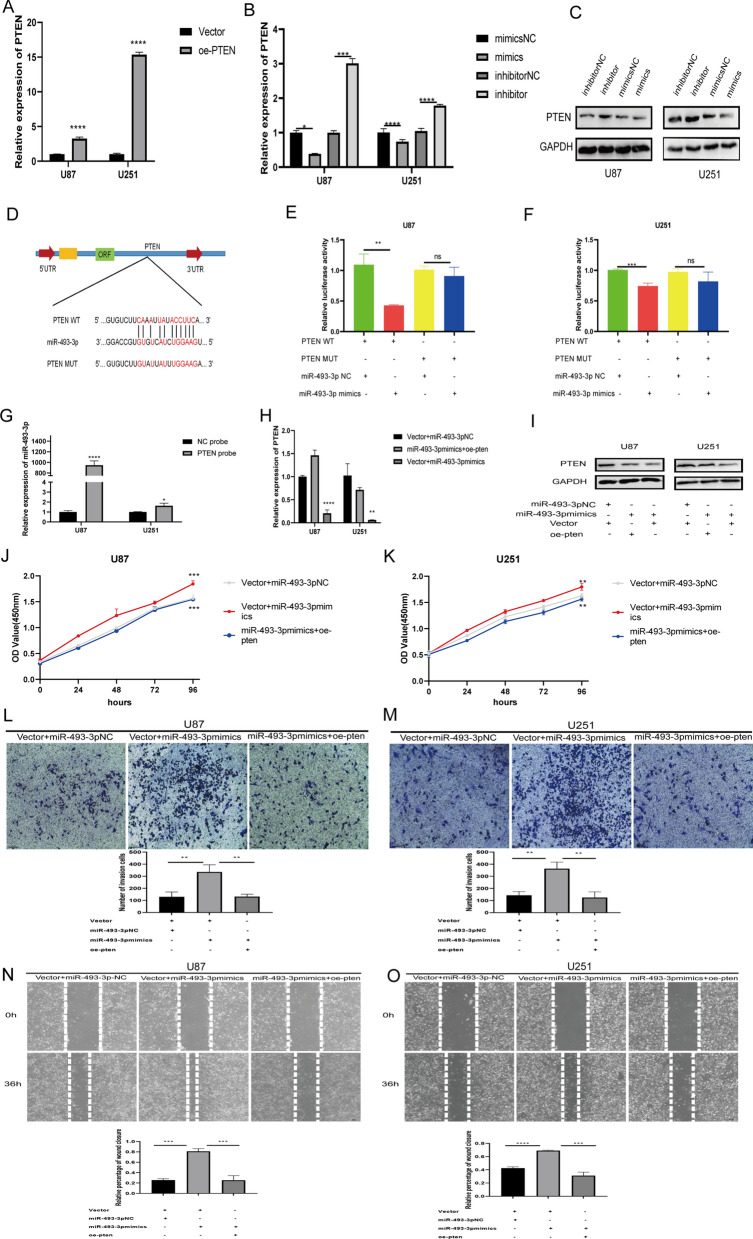
MiR-493-3p targeting PTEN promotes proliferation and migration of glioma cells in vitro. **A** qRT-PCR analyzed the expression of PTEN in U87 and U251 cells overexpressing PTEN. **B**, **C** Relative mRNA and protein levels of PTEN were evaluated by qRT-PCR (**B**) and western blot (**C**), respectively, in cells transfected with the miR-493-3p mimics and inhibitor. **D** Schematic illustration of PTEN-WT and PTEN-MUT luciferase reporter vectors. **E**, **F** After co-transfection with PTEN-WT or PTEN-MUT and miR-493-3p mimics or NC, the relative luciferase activity of U87 and U251 cells was detected. **G** RNA pulldown assay was executed in U87 and U251 cells, followed by qRT-PCR to detect the expression of miR-493-3p. **H**, **I** qRT-PCR (**H**) and western blot (**I**) were used to detect relative PTEN mRNA and protein levels, respectively, in U87 and U251 cells transfected with vector and miR-493-3p NC, vector and miR-493-3p mimics and miR-493-3p mimics and oe-PTEN. **J**, **K** CCK-8 was used to detect the proliferation abilities of U87 and U251 cells transfected with vector and miR-493-3p NC, vector and miR-493-3p mimics and miR-493-3p mimics and oe-PTEN. **G**, **H** The migration abilities of U87 and U251 cells transfected with vector and miR-493-3p NC, vector and miR-493-3p mimics, and miR-493-3p mimics and oe-PTEN were tested by wound healing test and Transwell assay. Scale bar: 100 µm. Data are expressed as the mean ± SD; ns indicates no significance. *P < 0.05, **P < 0.01, ***P < 0.001, ****P < 0.0001. Vector: empty vector control; NC: negative control oe-PTEN: PTEN overexpression vector; si-NC: negative control siRNA

Few studies have explored the role played by miR-493-3p in glioma. Therefore, we aimed to clarify the mechanism of action and biological functions of miR-493-3p in glioma cells. According to the prediction results from starBase V3, PTEN was suggested as a potential target gene of miR-493-3p. PTEN has been widely reported to act as a tumor suppressor [17, 18] and has been shown to inhibit tumor progression in gliomas [26]. Therefore, we conducted the next experiment to prove that PTEN binds to miR-493-3p. We constructed a PTEN overexpression vector, which was successfully expressed in U87 and U251 cells (Fig. 6A). To explore whether miR-493-3p regulates the expression of PTEN, miR-493-3p mimics and inhibitor were separately transfected into U87 and U251 cells. The results showed that the expression of PTEN decreased in both cell lines expressing the miR-493-3p, whereas PTEN expression increased in the miR-493-3p inhibitor groups (Fig. 6B, C), which suggested that miR-493-3p can affect the expression of PTEN in glioma cells.

PTEN-WT and PTEN-MUT were further cloned into the pmirGLO luciferase reporter vector and cotransfected in U87 and U251 cells, together with miR-493-3p mimics or NC mimics, to determine the interaction between miR-493-3p and PTEN (Fig. 6D). The results showed that the luciferase reporter activity in both cell lines decreased significantly in the PTEN-WT and miR-493-3p mimics group; however, these effects disappeared when the PTEN binding site was mutated. There were showed that PTEN binds to miR-493-3p (Fig. 6E, F). To confirm the predicted interaction, we performed a pulldown analysis using a biotinylated PTEN probe. In U87 and U251 cells, miR-493-3p was significantly enhanced after PTEN capture (in U87 cells, 1000-fold increase; in U251 cells, twofold increase; Fig. 6G).

In addition, we cotransfected U87 and U251 cells with PTEN overexpression vector or NC vector and miR-493-3p mimics to explore the biological effects of the miR-493-3p targeting of PTEN on glioma cells. The results of qRT-PCR and western blot analyses showed that the expression of PTEN in U87 and U251 cells was significantly decreased in the miR-493-3p mimics and NC vector groups, whereas the expression of PTEN in the miR-493-3p mimics and PTEN overexpression vector group was significantly higher than that in the miR-493-3p mimics and NC vector group (Fig. 6H, I).

We further explored the biological function of the miR-493-3p targeting of PTEN in glioma cells. The CCK8 growth curve showed that miR-493-3p mimics and NC vector group showed significantly increased cell proliferation activity, whereas the cell proliferation of the miR-493-3p mimics and PTEN overexpression vector group was lower than that of the miR-493-3p mimics and NC vector group (Fig. 6J, K). The effects of miR-493-3p on the migration of glioma cells were detected by wound healing and Transwell experiments. The results showed that the miR-493-3p mimics and NC vector group were characterized by the enhanced migration abilities of U87 and U251 cells, whereas the migration abilities of U87 and U251 cells decreased in the miR-493-3p mimics and PTEN overexpression group (Fig. 6L–O). These results suggested that miR-493-3p can promote the migration and proliferation of gliomas through the regulation of PTEN.

### CircNALCN acts as an miRNA sponge of miR-493-3p to regulate the expression of PTEN

**Fig. 7:**
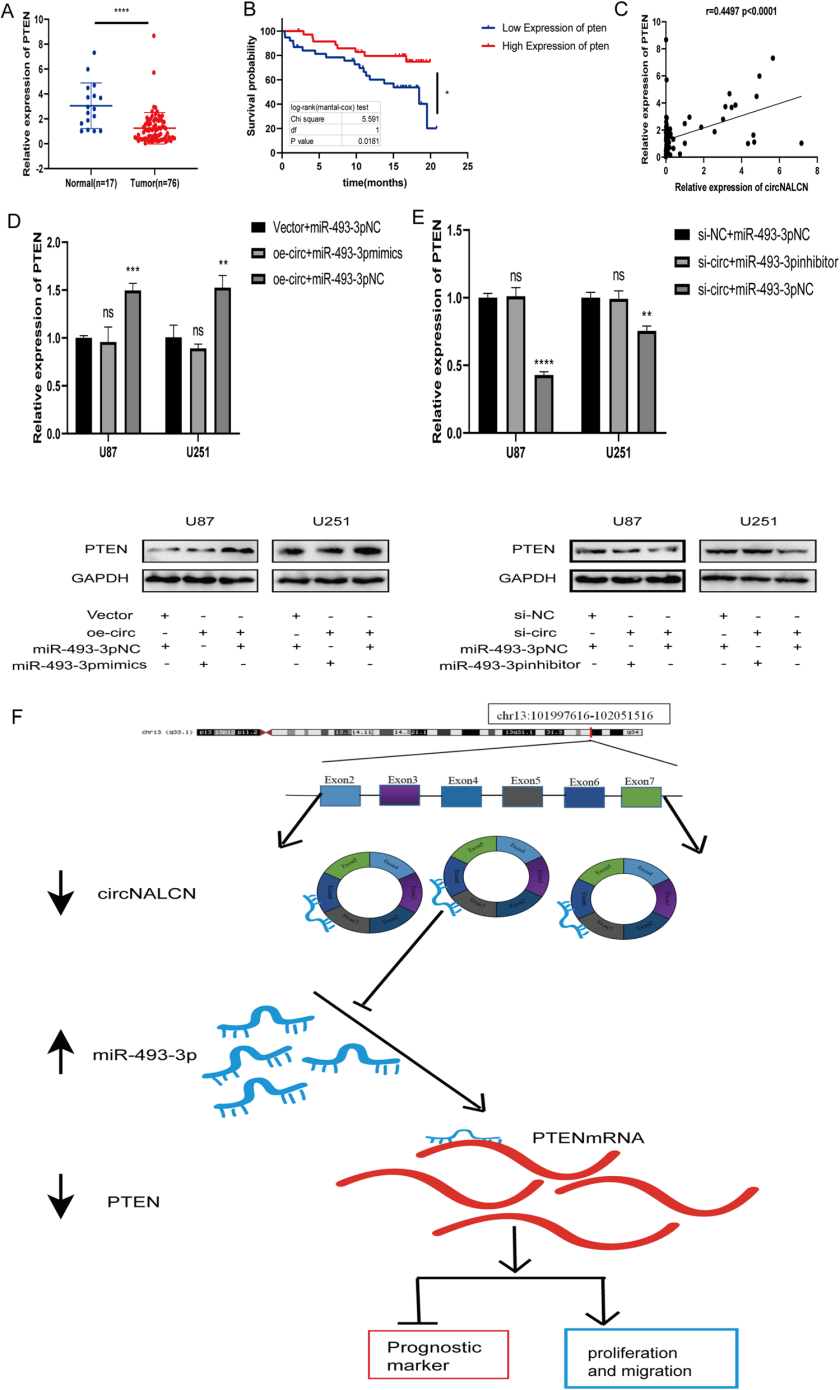
CircNALCN, as an miRNA sponge for miR493-3p, regulates the expression of PTEN. **A** qRT-PCR was used to detect the relative expression of PTEN in glioma tissues (n = 76) and normal brain tissues (n = 17). **B** Kaplan–Meier survival analysis showed that OS in glioma patients with the high expression of PTEN was higher than that in glioma patients with low PTEN expression. **C** Pearson’s correlation analysis of the expression of circNALCN and PTEN in brain glioma. **D**, **E** The relative mRNA and protein levels of PTEN were detected by qRT-PCR and western blot, respectively, in U87 and U251 cells transfected with vector and miR-493-3pNC, oe-circNALCN and miR-493-3p mimics, oe-circNALCN and miR-493-3pNC, si-NC and miR-493-3p NC, si-circNALCN and miR-493-3p inhibitor, and si-circNALCN and miR-493-3p NC. ns indicates no significance. **F** The diagrammatic representation of our conclusion was shown. *P < 0.05, **P < 0.01, ***P < 0.001, ****P < 0.0001. Vector: empty vector control; NC: negative control oe-circNALCN: circNALCN overexpression vector; si-NC: negative control siRNA; si-circNALCN: circNALCN siRNA

To further explore the interaction among circNALCN, miR-493-3p and PTEN, we performed a series of experiments. First, we detected the expression of PTEN in glioma and normal tissues. The qRT-PCR results showed that the expression of PTEN in glioma tissues was significantly lower than that in normal tissues (Fig. 7A), and the Kaplan–Meier curve showed that the expression level of PTEN was positively correlated with OS among glioma patients (Fig. 7B), which was consistent with the results of previous research [26]. We found a positive correlation between the expression of circNALCN and PTEN in both gliomas and normal tissues (Fig. 7C). In addition, qRT-PCR and western blot assays demonstrated that the overexpression of circNALCN significantly increased the mRNA and protein levels of PTEN, whereas the co-transfection of the circNALCN overexpression vector with miR-493-3p mimics appeared to counteract these effects in U87 and U251 cells (Fig. 7D). Similarly, the knockdown of circNALCN significantly decreased the mRNA and protein levels of PTEN, whereas the co-transfection of si-circNALCN and miR-493-3p inhibitor eliminated these effects in U87 and U251 cells (Fig. 7E). These data indicated that circNALCN acts as a ceRNA for miR-493-3p to regulate the expression of PTEN, inhibiting the occurrence and development of gliomas.

## Discussion

Increasing evidence regarding the biogenesis and functions of circRNAs has provided a new perspective for understanding the pathogenesis of a variety of diseases, including cancer, cardiovascular diseases, and autoimmune diseases [27–29]. Some circRNAs are thought to play oncogenic or tumor-suppressive roles in various human cancers, such as colon cancer, lung cancer, hepatocellular carcinoma (HCC), and gastric cancer [30]. Moreover, the mechanisms of certain glioma-related circRNAs have gradually been revealed [31]. However, this study report the role played by the circNALCN/miR-493-3p/PTEN pathway in the occurrence and development of gliomas.

First, we identified a new circular RNA, named circNALCN, through a high-throughput RNA-seq analysis. CircNALCN was found to be significantly downregulated in gliomas and significantly correlated with tumor grade and OS in glioma patients. Second, through circRNA functional tests, we confirmed that circNALCN overexpression could inhibit the proliferation and migration of glioma cells. Third, the binding of circNALCN to miR-493-3p was predicted by bioinformatics analysis and verified by double-luciferase reporting assay, FISH analysis, and pulldown experiments. We also found that miR-493-3p was significantly upregulated in glioma cells and tissues and correlated with OS and tumor grade in patients. Fourth, mechanistic experiments demonstrated that circNALCN acts as an miR-493-3p sponge to counteract the miR-493-3p-mediated inhibition of the target gene PTEN during glioma progression. Together, these data suggested that circNALCN may serve as a new biomarker and therapeutic target, with an important role in glioma progression and development.

Most circRNAs contain miRNA response elements (MREs), and studies have increasingly shown that the ceRNA mechanism represents the primary mechanism through which circRNAs affect biological functions [25]. Using circBank, starBase, and high-throughput sequencing prediction analysis, circNALCN was predicted to contain an miRNA binding sites for miR-493-3p. Therefore, FISH, biotin-labeled probe pulldown, and double-luciferase reporter gene assays were used to confirm that circNALCN and miR-493-3p colocalized in the cytoplasm of glioma cells and bound directly. Subsequent rescue experiments further confirmed that miR-493-3p could reverse the antitumor effects of circNALCN. Therefore, our results provide evidence that circNALCN acts as a sponge for miR-493-3p, providing a developable biomarker and therapeutic target for glioma patients.

PTEN was identified as a target gene of miR-493-3p by bioinformatics analysis, which was confirmed by luciferase reporter gene analysis. In gliomas, miR-492-3p negatively regulates the expression of PTEN, whereas circNALCN positively regulates the expression of PTEN. Previous studies have shown that PTEN acts as a tumor suppressor in most human tumors, such as pancreatic cancer, HCC, and breast cancer [32–34]. Decreased PTEN expression is typically associated with aggressive tumors and a worse prognosis. An abnormal decrease in PTEN expression is considered to serve as a predictor of malignant progression in gliomas, and PTEN can be used as a marker to predict the prognosis of patients with gliomas [26]. Previous studies have found that PTEN participates in the negative regulation of phosphatidylinositol-triphosphate (PIP3) and plays a tumor inhibitory role by negatively regulating AKT signal transduction [35]. Recent studies have shown that circFBXW7 plays a tumor inhibitory role by reversing the expression of NEK2 and mammalian target of rapamycin (mTOR), promoting the expression of PTEN in tumors [36]. Our results showed that the expression of PTEN is low in gliomas and is associated with the OS of patients with gliomas. To date, whether and how circNALCN is involved in the progression of gliomas induced by PTEN remains unclear. Our results in this study showed that the interaction between circNALCN and miR-493-3p promotes the expression of PTEN, suggesting a potential mechanism through which the circNALCN/miR-493-3p/PTEN axis regulates the progression of glioma.

We studied the expression, function and clinical significance of circNALCN in glioma and researched on the relationship between miR-493-3p and PTEN. These findings may have implications for the treatment of gliomas. However, the interpretation of our research results has several limitations. Firstly, the glioma tissue used for the RNA-seq analysis in this study was taken from a homologous population at a hospital, and we cannot rule out the possibility that other important downregulated circRNAs may also be involved in the progression of PTEN-induced gliomas. Secondly, our preliminary study showed that the expression of circNALCN in glioma tissue was significantly downregulated, and up-regulation of circNALCN can inhibit the proliferation and migration of glioma cells. However, we only performed functional tests of circNALCN in vitro. Additional animal experiments are being performed to further explore the tumor inhibitory effect of circNALCN in vivo. Thirdly, our study confirmed the ability of circNALCN to combine with miR-493-3p. However, other miRNAs may also bind circNALCN to regulate the progress and development of gliomas. Fourth, whether circNALCN regulates the progression of gliomas through other mechanisms, such as interactions with RBPs or the sponge absorption of trans-acting elements, requires further study. Therefore, a more in-depth understanding of the therapeutic potential of circNALCN in gliomas remains necessary.

## Conclusion

In summary, our results provide evidence to support the downregulation of circNALCN expression in gliomas, which may play a role as a tumor suppressor and prognostic biomarker in gliomas. In addition, we also indicated that circNALCN inhibits the proliferation and migration of glioma cells by downregulating miR-493-3p and upregulating the expression of PTEN. Our results demonstrate the potential mechanism through which circNALCN may regulate the progression of glioma cells through the circNALCN/miR-493-3p/PTEN axis, providing a new potential therapeutic target for glioma patients.

## Supplementary Information

**Additional file 1: Table S1.** FISH probe for circNALCN and miR-493-3p.

## Data Availability

The data sets used and analyzed during the current study are available from the corresponding author on reasonable request.

## Abbreviations

WHO: World Health Organization
PTEN: Phosphatase and tensin homolog
circRNAs: Circular RNAs
circNALCN: CircRNA NALCN
miR-493-3p: MicroRNA-493-3p
FBS: Fetal bovine serum
qRT-PCR: Quantitative real-time polymerase reaction
GAPDH: Glyceraldehyde 3-phosphate dehydrogenase
si-circNALCN: CircNALCN small interfering RNA
NC: Negative control
RNA-seq: RNA sequencing
RBPs: RNA binding proteins
MiRNAs: MicroRNAs
IGF2BP3: Insulin-like growth factor 2 mRNA binding protein 3
STAT3: Signal transducer and activator of transcription 3
DMEM: Dulbecco’s modified Eagle medium
gDNA: Genomic DNA
TAE: Tris-acetate-EDTA
FISH: Fluorescence in situ hybridization
FAM: Fluoresceine amidite
Cy3: Cyanine-3
RAP: RNA antisense purification
OD: Optional density
OS: Overall survival
FC: Fold change
DEcircRNAs: Differentially expressed circRNAs
