# Supplementary material for: CircRNA NALCN acts as an miR-493-3p sponge to regulate PTEN expression and inhibit glioma progression
Source: Cancer Cell Int. 2021 Jun 10;21:307. doi: 10.1186/s12935-021-02001-y (PMC8194043; doi:10.1186/s12935-021-02001-y)
Supplement: Supplementary file 1 — Additional file 1: Table S1. FISH probe for circNALCN and miR-493-3p. [file 12935_2021_2001_MOESM1_ESM.docx]

|  | Name | Sequence |
| --- | --- | --- |
| FAM | CircNALCN(hsa_circ_0099761) | GCACAAAACCACAGTCTATCTCATTAAAGCCA |
| Cy3 | hsa-miR-493-3p | CCTGGCACACAGTAGACCTTCA |

**Table s1**

**FISH probe for circNALCN and miR-493-3p**
